# Structural and optical characterization of metal tungstates (MWO_4_; M=Ni, Ba, Bi) synthesized by a sucrose-templated method

**DOI:** 10.1186/1752-153X-7-80

**Published:** 2013-05-01

**Authors:** Siti Murni M Zawawi, Rosiyah Yahya, Aziz Hassan, H N M Ekramul Mahmud, Mohammad Noh Daud

**Affiliations:** 1 Department of Chemistry, Faculty of Science, University of Malaya, Kuala Lumpur 50603, Malaysia

**Keywords:** Metal tungstates, Sucrose, Optical

## Abstract

**Background:**

Metal tungstates have attracted much attention due to their interesting structural and photoluminescence properties. Depending on the size of the bivalent cation present, the metal tungstates will adopt structures with different phases. In this work, three different phases of metal tungstates MWO_4_ (M= Ba, Ni and Bi) were synthesized via the sucrose templated method.

**Results:**

The powders of BaWO_4_ (tetragonal), NiWO_4_ (monoclinic) and Bi_2_WO_6_ (orthorhombic) formed after calcination temperatures of 750, 650 and 600°C for 4 h respectively are found to be crystalline and exist in their pure phase. Based on Scherrer estimation, their crystallite size are of nanosized. BET results showed NiWO_4_ has the highest surface area. BaWO_4_ exhibited less Raman vibrations than the NiWO_4_ because of the increased lattice symmetry but Bi_2_WO_6_ showed almost the same Raman vibrations as BaWO_4_. From the UV-vis spectra, the band gap transition of the metal tungstates are of the order of BaWO_4_ > Bi_2_WO_6_ > NiWO_4_. Broad blue-green emission peaks were detected in photoluminescence spectra and the results showed the great dependence on morphology, crystallinity and size of the metal tungstates.

**Conclusion:**

Three different phases of metal tungstates of BaWO_4_ (scheelite), NiWO_4_ (wolframite) and Bi_2_WO_6_ (perovskite layer) in their pure phase were successfully prepared by the simple and economical sucrose-templated method. The highest surface area is exhibited by NiWO_4_ while largest band gap is shown by BaWO_4_. These materials showed promising optical properties.

## Introduction

Metal tungstates with formula MWO_4_ have attracted much attention due to their interesting structural and photoluminescence properties [1-5]. These materials have found applications in scintillation counters, lasers and optical fibers [6,7]. Some of the divalent transition metal tungstates have also gained commercial interest in lasers and fluorescent lamps, while some are of special importance due to their electrical conductivity and magnetic properties. In addition, these materials also find applications as catalysts and humidity sensors [8,9].

In the MWO_4_ compounds, if M^2+^ has small ionic radius < 0.77 Å (Ni = 0.69), it will belong to the wolframite-type monoclinic structure where the tungsten atom adopts an overall six-fold coordination [10]. However, if larger bivalent cations with ionic radius > 0.99 Å (Ba=1.35), they exist in the so-called scheelite-type tetragonal structure where the tungsten atom adopts tetrahedral coordination. Bismuth tungsten oxide belongs to the orthorhombic system, space group P*ca*2_1_, and crystallizes in a layered crystal structure including the corner-shared WO_6_. The Bi atom layers are sandwiched between WO_6_ octahedral layers [11]. It is the simplest member of the Aurivillius family from Bi_2_A_n-1_B_n_O_3n+3_ (A=Ca, Sr, Ba, Pb, Bi, Na, K and B=Ti, Nb, Ta, Mo, W, Fe) (when n=1) of layered perovskites, which structurally comprises of alternating perovskite-like slabs of WO_6_ and [Bi_2_O_2_]^2+^ layers. Recently, many studies have been reported on the preparation and characterization of metal tungstates using various preparation methods such as Czochralski [12], precipitation [13,14], hydrothermal [11,15], solid state [16], pulsed laser deposition [17]. Meanwhile the nanostructures of metal tungstates in different crystal structures including nanorods, nanoparticles, hollow clusters and others have been prepared by chemical and physical methods. For Bi_2_WO_6_, its nanometer sheet shaped was obtained through hydrothermal treatment at pH=11, heated at 200°C for 24 hours and finally thermally treated at 400, 600 and 800°C for 3 hours [18]. BaWO_4_ in the rhombic shape was prepared by a molten flux reaction using alkali metal nitrates as the reaction media [19]. Nickel tungstate (NiWO_4_) nanoparticles were successfully synthesized at low temperatures by a molten salt method at a temperature as low as 270°C, where the mixture of NaNO_3_ and LiNO_3_ was used as the molten salt medium with 6:1 mass ratio of the salt to the NiWO_4_ precursor [20]. Generally, these methods require expensive and sophisticated equipment, high temperatures with long processing times, expensive precursors and high consumption of electric energy.

Prabhakaran et al. [21] had used a cheaper and simpler method of using sucrose in order to synthesize yttria-stabilized zirconia (YSZ) nanoparticles in both acidic and basic solutions. The analyses consistently reported to have fairly uniform nanoparticles with small size, containing both tetragonal and monoclinic phases with crystallite size between 10 and 30 nm. Due to its simplicity, the sucrose-template method has great potential for manufacturing high quality ultrafine ceramic oxides economically [22] and this creates a new approach for synthesis of the other ceramic materials. In this method, the -OH and -COOH groups of the decomposed sucrose products help in binding the metal ions in the homogeneous solution, which reduces the chances of precipitation. During the decomposition process, a voluminous, organic-based, black, fluffy mass of carbonaceous material is formed which upon heating will decompose further into carbon dioxide and water and a large amount of heat is generated. The outgoing gases prevent agglomeration, and form pores and fine particles with high surface area in the final products. The aim of this paper is to synthesize the different crystal structures of BaWO_4_, NiWO_4_ and Bi_2_WO_6_ by a sucrose templated method and to characterize the materials for their structural and optical property by X-ray Diffraction (XRD), Field Emission Scanning Electron Microscopy (FESEM), Brunaer-Emmet-Teller (BET) and Raman spectroscopy while optical properties were investigated using UV-vis and photoluminescence spectroscopy.

### Experimental details

#### Preparation of powders

The desired metal nitrates [Ba(NO_3_)_2_, Ni(NO_3_)_2_∙ 6H_2_O, Bi(NO_3_)_2_] of 2.6135, 2.9081 and 4.8511 g were individually dissolved in distilled water before being mixed into an aqueous solution of sucrose. This is followed by addition of an equal volume of 2.4633 g of ammonium metatungstate to maintain stoichiometric ratio (1:1) with continuous stirring. Sucrose acts as a template and the ratio of sucrose to metal used was 3:1. Towards the end of the evaporation, the precursor solution (after further heating) gave rise to a fluffy black organic mass. The carbon rich mass was easily crushed to form the precursor powders. Precursor powders are denoted as MW*p* (M= Ba, Ni, and Bi). Calcination treatment was applied in the next step because of the large amount of organic compounds present in the crunchy powders. The temperatures and durations for calcinations were derived from the results of the thermogravimetric analysis whereby processes such as dehydration and other volatilizations to go to completion before proceeding to higher temperatures.

The calcination treatment applied to the samples involved heating at the rate of 10°C/min and the temperature was held constant for 4 h for each thermal change as inferred from the thermal analysis to allow completion of each of the processes. The three powdered precursors, MW*p* (M= Ba, Ni, and Bi) were subsequently calcined at 750, 650 and 600°C respectively for 4 h and the samples were denoted as MWO_4_ (M= Ba, Ni, and Bi).

#### Characterization

The formation of oxides was monitored by X-ray diffraction (XRD) measurements using Siemen D5000 with a copper K_α_ radiation tube and wavelength λ of 1.54 Å, operated at 40 kV and 40 mA. The X-ray powder diffraction patterns were obtained in the range 5-60°, with increments of 0.05°. The crystalline phases were identified by using the International Centre for Diffraction Data (ICDD). The full width at half maximum (FWHM) of the diffraction peaks obtained from the refinement have been used to calculate the crystallite size. Specific surface area (S_BET_) measurements were made with a Quantachrome AUTOSORB-1 model by nitrogen adsorption at -196°C using the BET isotherm. Samples were degassed under flowing argon at 250°C for 9 h before being adsorbed by nitrogen. The surface morphology of the samples was e analyzed using the Field Emission Scanning Electron Microscope, FESEM JSM-7500F/7500FA (JEOL) at magnification of 20,000 ×. This morphological analysis can provide information on the prevalent surface features. FESEM images allowed us to estimate the average particle size distribution of all three samples through the counting of approximately 150 particles using Image tool software. Diffuse reflectance spectra were obtained using a UV-Visible Spectrophotometer (Shimadzu). Raman spectra was collected by InVia Raman Microscope Renishaw spectrometer using UV lens set at λ_UV_ = 325 nm and equipped with 2,400 l/nm diffraction grating. The same equipment was also used for photoluminesence (PL) analysis by using a visible lens set and equipped with 1,200 l/nm diffraction grating.

## Results and discussion

### XRD

**Figure 1:**
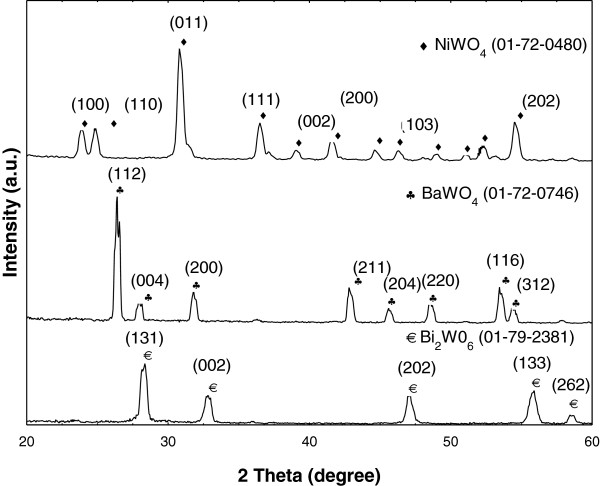
**The XRD patterns of NiWO**_**4**_**, BaWO**_**4 **_**and Bi**_**2**_**WO**_**6 **_**samples.** All samples were synthesized by sucrose template method.

XRD pattern can reveal the phase purity and crystallinity of the powder sample. Figure 1 shows sharp diffraction peaks indicating that the oxide products are well crystallized and no peaks attributable to other impurities were observed. The pattern agrees well with the JCPDS file of NiWO_4_, BaWO_4_ and Bi_2_WO_6_ (PDF card 72-0480, 72-0746 and 79-2381). The NiWO_4_ indexed in wolframite monoclinic structure (space group: *P*2/c, with Z = 2) is characterized by alternating layers of transition-metal and tungsten atoms parallel to the (100) plane. The oxygen atoms are hexagonally closely packed and the metal ions occupy a quarter of all the octahedral sites [23]. For BaWO_4_, the peaks from diffraction patterns are consistent with a body-center primitive tetragonal scheelite, space group I4_1_/a and has $C_{4h}^{6}$ point group with two formula units per primitive cell. In an ideal scheelite type of ABO_4,_ larger A (Ba^2+^) cation shows eight-fold coordination and smaller B (W^6+^) cation shows four-fold coordination. The tungstates reported have strong covalent bonds of W-O in [WO_4_]^2-^ molecular ionic units and weak coupling between [WO_4_]^2-^ anions and Ba^2+^ cations [24]. All the peaks of Bi_2_WO_6_ are recognized with the crystal structure of orthorhombic symmetry crystal phase with space group *Pca*2_1_ and crystallized in a layered crystal structure including the corner-shared WO_6._ The Bi atom layers are sandwiched between WO_6_ octahedral layers [11].

Table 1 shows that crystallite sizes of all the samples calculated from Scherer’s equation are in nano-size range: NiWO_4_ at d_100_, d_110_ and d_011_ are 19.3, 19.3 and 17.2 nm, while those of BaWO_4_ at d_112_ and d_004_ are 18.9 and 17.4 nm. Smaller crystallite sizes of 15.5 and 14.9 nm are shown by Bi_2_WO_6_ at d_131_ and d_002_, respectively. Dong Young et al. [18] have also synthesized similar compounds by commercial hydrothermal methods and obtained the crystallite size of 17-24 nm in same plane of d_131_. NiWO_4_ synthesized by reacting ammonium metatungstate and nickel nitrate as a function of temperature from 673 to 1073 K of 1 h reaction time has been reported by Quintana-Melgoza et al. [25] and average crystallite size as determined by Scherrer analysis obtained was from 55 to 112 nm, which is three times bigger than that reported in this work. In the case of BaWO_4_, calculated crystallite size synthesized by room temperature the metathetic reaction method has been reported to grow twofold in crystallite size (51 nm) along d_112_[26]. The existence of sucrose in the solution of the metal cations will form a matrix in which the metal cations are distributed through the sucrose structure. The sucrose molecule is hydrolyzed into glucose and fructose and in this way sugar recrystallization is prevented. The complex mass is obtained by complexation via gel formation and the final particles are obtained upon decomposition in the calcination process. During heating, the metal ion complex is decomposed into CO_2_ and H_2_O and a large amount of heat is generated. All these products are gaseous, preventing agglomeration and thus giving rise to pores and fine powders of smaller crystallite size (Table 1).

**Table 1:** Summary of metal tungstates phase formation and calculated crystallite sizes

**System**	**X-ray phase**	**Crystal structure**	**2Θ**	**hkl**	**t (nm)**
NiWO_4_	Wolframite	Monoclinic	23.89	100	19.3
			24.85	110	19.3
			30.87	011	17.2
BaWO_4_	Scheelite	Body cubic tetragonal	26.38	112	18.9
			27.99	004	17.4
Bi_2_WO_6_	Orthorhombic	Orthorhombic	28.29	131	15.5
			32.83	002	14.9

#### FESEM and BET

**Figure 2:**
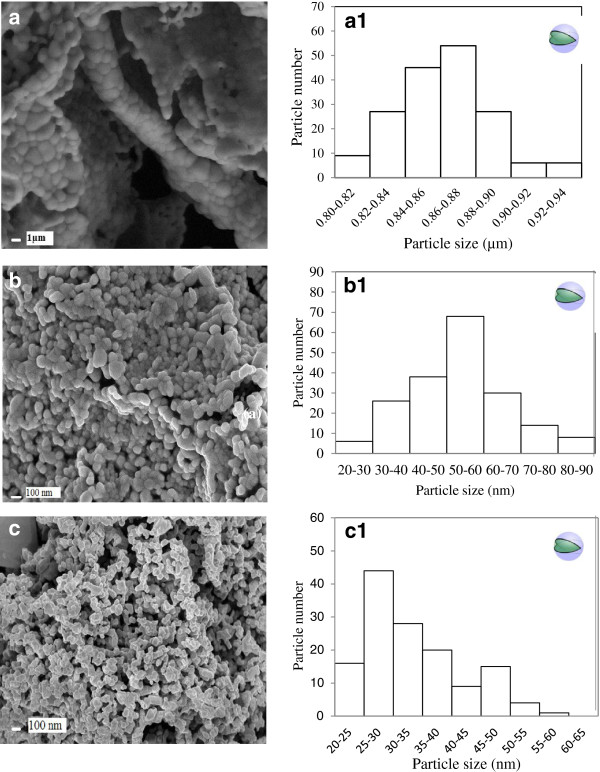
**FESEM micrographs the metal tungstates and the calculated particle size distributions.** BaWO_4_ (**a**) (a1), NiWO_4_ (**b**) (b1), Bi_2_WO_6_ (**c**) (c1).

**Figure 3:**
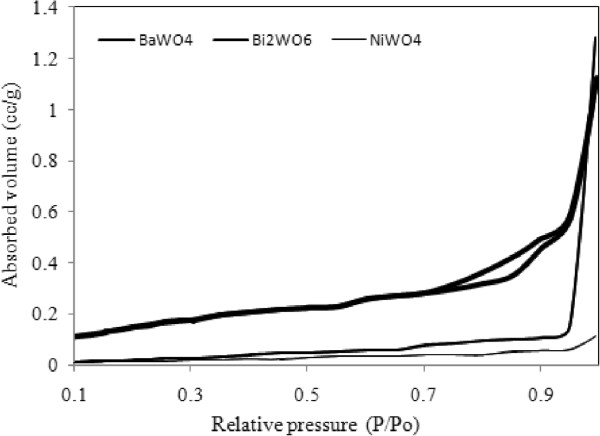
**N**_**2 **_**adsorption isotherms of BaWO**_**4**_**, Bi**_**2**_**WO**_**6 **_**and NiWO**_**4 **_**samples.**

The FESEM results demonstrated that the morphology of BaWO_4_, NiWO_4_ and Bi_2_WO_6_ samples strongly depend on size of particles while BET results showed the dependency of their surface areas on pore volume and pore distribution. All three samples show different morphologies: BaWO_4_ particles (Figure 2(a)) grow in large spherical grain sizes between ~0.8-0.9 μm. Samples NiWO_4_ and Bi_2_WO_6_ in Figure 2(b & c) show smaller inter-connected grain sizes of 30-90 and 20-60 nm, respectively. From Figure 3, BaWO_4_ shows mesoporous characteristics obtained from adsorption-desorption isothermal of type IV and the H3 and the hysteresis loop observed in the range of 0.70 – 0.95 P/P_o_ (according to the IUPAC classification) agrees reasonably well with the small pore volume (0.05 cm^3^g^-1^) and low surface area (2.30 m^2^g^-1^), as shown in Table 2. Both samples of NiWO_4_ and Bi_2_WO_6_ show adsorption-desorption isotherms of a macroporous characteristic (type III) with absence of any hysteresis loop.

**Table 2:** Summary of metal tungstates specific surface area, pore volume and pore size distribution

**Sample**	**S**_**BET **_**(m**^**2**^**g**^**-1**^**)**	**Pore volume (cm**^**3**^**/g)**	**Pore size distribution, (nm)**
Bi_2_WO_6_	3.58	0.62	1.54
NiWO_4_	20.06	0.54	2.46
BaWO_4_	2.30	0.05	1.92

Even though the NiWO_4_ sample has larger crystallite size (according XRD), its surface area is fivefold larger (20.06 m^2^g^-1^) than Bi_2_WO_6_ (3.58 m^2^g^-1^). This phenomenon is attributed to the higher pore distribution (Table 2) and less agglomeration of NiWO_4_ itself (Figure 2(b)). This finding shows that the prepared NiWO_4_ sample using sucrose solution evaporation has higher BET surface area compared to NiWO_4_ synthesized by combustion method (< 11 m^2^g^-1^) even though a spherical-like morphology was obtained in both cases [27]. Bi_2_WO_6_ synthesized by using co-precipitation method also resulted in similar spherical particles (after calcinations at 600, 700 and 800°C) as reported by Alfaro and de la Cruz [28], but the size of particles were in microns; sizes (~1-2 μm) and BET values obtained were 0.3- 1.5 m^2^g^-1^, which was 10 times lower than the findings in this work shown in Table 2.

FESEM images can also allow the estimation of the average particle size distribution of samples by counting approximately 150 particles using an Image tool software. The particles are assumed spherical-like (Figure 2(a1-c1)). Figure 2(a1) shows the average particle size distribution (diameter) in the range from 0.80-0.94 μm for BaWO_4_. The figure shows that 59% of the particles with a spherical-like morphology presented an average area of 0.84-0.88 μm. Figure 2(b1) shows the average particle area distribution of Bi_2_WO_6_ is 20-55 nm and that 62% of the particles presented an average area of 25-35 nm, smaller than BaWO_4_ (in micron range). As for NiWO_4_ (Figure 2c1) with the plate-like morphology the average particle area distribution is 30-90 nm, which is in close relationship with the above grain size (FESEM image). These results show that the sucrose-templated method is able to influence the growth process into nano-range for samples NiWO_4_ and Bi_2_WO_6_, except for sample BaWO_4_ which is in micron size. However, the particle size distribution of BaWO_4_ synthesized using a sucrose-templated method shows smaller dimension (0.84-0.88 μm) compared to BaWO_4_ synthesized by co-precipitation followed by domestic microwave-hydrothermal at 413 K for different times which resulted in a large self-assembled microcrystal of height (0.30–11.85 μm) and width (0.25–2.30 μm) [29].

#### Raman spectra

**Figure 4:**
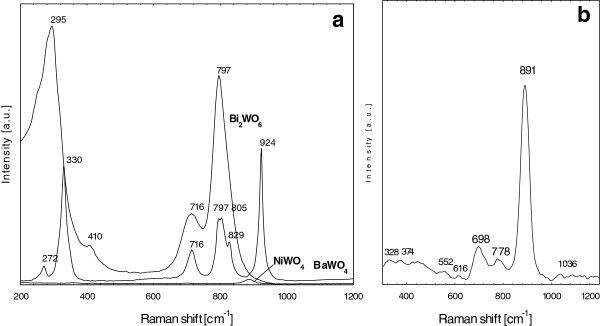
**Raman spectra for the metal tungstates.** (**a**) NiWO_4_, BaWO_4_ and Bi2WO (**b**) enlarged spectra for NiWO_4_.

The structural order at short-range for the three different phases of NiWO_4_, BaWO_4_ and Bi_2_WO_6_ nanoparticles was determined by Raman active phonon modes (Figure 4). Hardcastle et al. [30], using the diatomic approximation method, concluded that for an ideal WO_4_ unit, the shortest W-O bond should correspond to a Raman fingerprint located at 874 cm^-1^ (νs(W=O)), with a standard deviation of approximately 55 cm^-1^.

Considering the Raman active modes of scheelite-type AWO_4_ compounds, there are two types of vibration modes, belonging to the internal and external vibrations. The first corresponds to the normal motion of atoms inside the [WO_4_]^2-^ tetrahedrons, and the second involves the vibration WO_4_ tetrahedrons against the divalent A atoms. Group theory calculation predicts 26 vibration modes for the tetragonal scheelite (BaWO_4_) primitives’ cell at wavevector k=0, which can be represented in (1) [31]:

(1) $$Г=3A_{g}+5A_{u}+5B_{g}+3B_{u}+5E_{g}+5E_{u}$$

where all 13 vibrations A_g_, B_g_ and E_g_ are Raman-active. As shown in Figure 4, the tetragonal BaWO_4_ has two strong vibrations at 924 and 330 cm^-1^ and four weak vibrations at 829, 797, 716 and 272 cm^-1^. It is predicted to have less Raman vibrations when compared to monoclinic NiWO_4_ because of the increased lattice symmetry. The two strong vibrations of 924 and 330 cm^-1^ and weak mode at 797 cm^-1^ can be assigned to the W-O stretching vibration of WO_4_ tetrahedra. The medium mode at 272 cm^-1^ is derived from symmetric stretching vibration of the BaO_6_ octahedra. All these modes are characteristic of the tetragonal scheelite structure as reported previously [32-36]. However, in our samples, the vibrations were slightly shifted and some vibration modes were not detected. These observations can be attributed to some differences in their geometries, particle sizes and nature of the products.

Comparatively, Raman vibrations for monoclinic wolframite structure would be expected to give six internal stretching modes caused by each of the six W-O bonds in the WO_6_ octahedrons and from group theoretical analysis of the monoclinic (NiWO_4_) yields 36 lattice modes [15]:

(2) $$Г=8A_{g}+10B_{g}+8A_{u}+8B_{u}$$

Here, 18 even (g) vibrations are Raman-active modes. As for monoclinic NiWO_4_, the corresponding spectrum in Figure 4 shows only three strong vibrations at 891, 778 and 698 cm^-1^ and five weak vibrations at 328, 374, 552, 616 and 1036 cm^-1^ corresponding to the normal W-O vibration of the WO_6_ octahedra. Unlike the ideal WO_4_ structure (scheelite) where four normal vibrational modes of the tetrahedral structure are Raman active, WO_6_ structure has six normal modes of vibration of which only three are Raman active. The isolated WO_6_ wolframite structure found in the bulk crystalline NiWO_4_ has 891 cm^-1^ which is associated with the WO_6_ symmetric stretching vibration and this agrees well with the results reported by Ross-Medgaarden and Wachs [14].

The factor group analysis predicts that there should be 105 optical modes for P*ca*2_1_ structure of Bi_2_WO_6_ distributed among 26A_1_ + 27A_2_ + 26B_1_ + 26B_2_ irreducible representations. The A_1_, B_1_ and B_2_ modes are both Raman and IR active whereas the A_2_ modes are only Raman active. Bi_2_WO_6_ shows two strong peaks at 797 and 295 cm^-1^ and weak peaks at 410 and 716 cm^-1^. The strongest peak at 797 cm^-1^ can be assigned to the symmetric and asymmetric stretching modes of the WO_6_ octahedra involved in the motions of the apical oxygen atoms perpendicular to the layer [30]. The weak Raman peak at 716 cm^-1^, is due to asymmetric stretching mode of the WO_6_ octahedra, involving mainly vibrations of the equatorial oxygen atoms within layers. The peak at 295 cm^-1^ region originates from the bending mode of the bismuth-oxygen polyhedral.

#### Diffuse reflectance UV-visible spectroscopy

**Figure 5:**
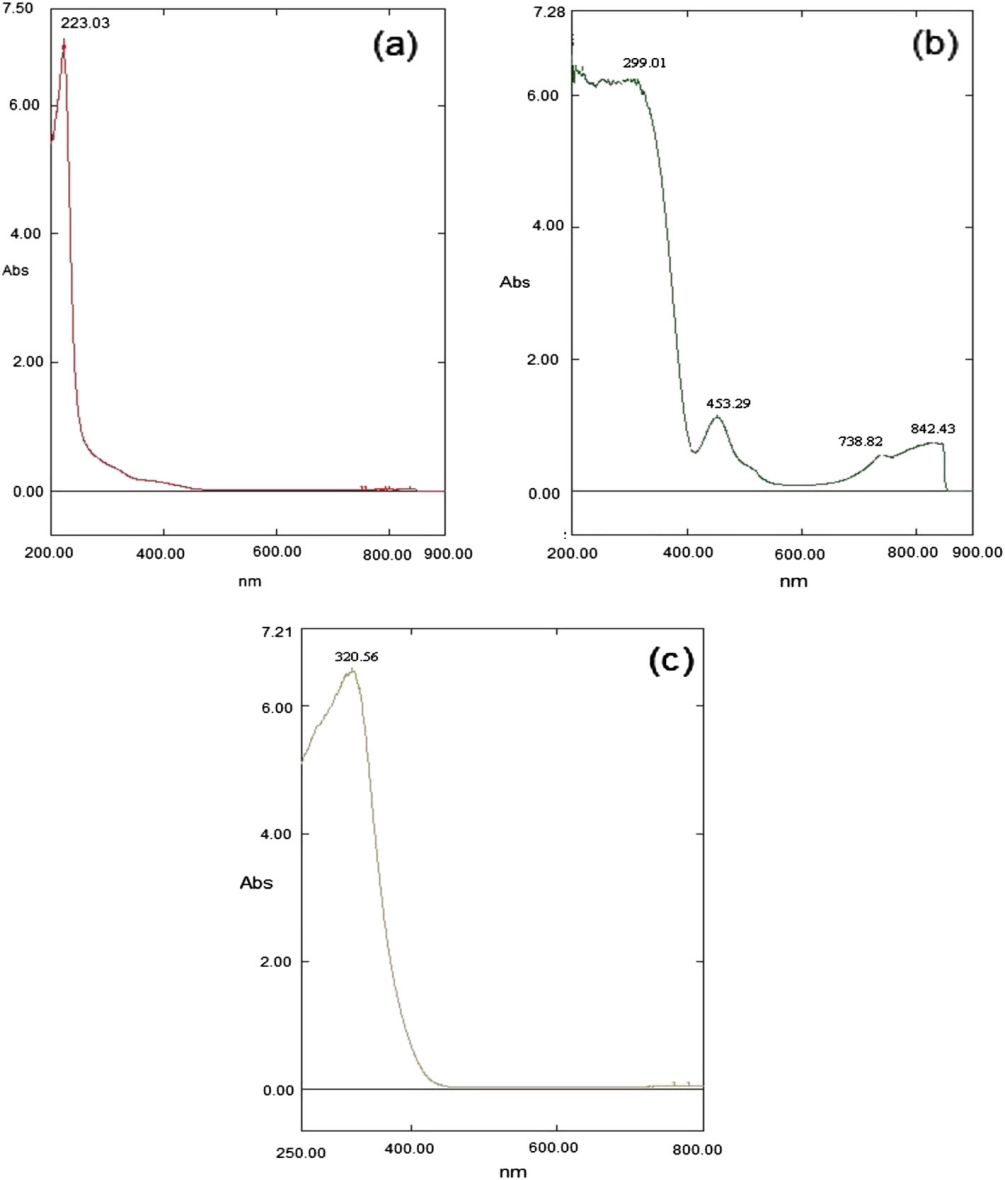
**Optical absorbance spectra of the metal tungstates.** (**a**) BaWO_4_, (**b**) NiWO_4_ and (**c**) Bi_2_WO_6_.

Figure 5 shows the optical absorption spectra of BaWO_4_, NiWO_4_ and Bi_2_WO_6_ nanoparticles with an absorption edge in 200–900 nm region. All samples have excellent optical transmission spectra as the maximum absorption edges appeared in the ultraviolet region: 223.0 nm for BaWO_4_, 320.6 nm for Bi_2_WO_6_ and 299.0 nm for NiWO_4_. The excitation from O_2p_ to Wt_2g_ in the (WO_4_^2-^) group absorbs ultraviolet irradiation in MWO_4_. In the excited state of the (WO_4_^2-^) groups, the hole (on the oxygen) and the electron (on the tungsten) remain together as an exciton because of their strong interactions. Further absorption peaks in the visible region are exhibited by NiWO_4_ which could be due to a charge transfer transition in which an oxygen 2*p* electron goes into one of the empty tungsten 5*d* orbital.

A unique feature of UV-vis for the isolated WO_4_ reference compounds is that they only possess a single ligand to metal charge transfer (LMCT) band in the general region of 218-274 nm, with many of the band maxima occurring at 220-250 nm. The exact location of this band maximum depends on the extent of distortion of the isolated WO_4_ structure [14]. Optical absorbances of samples BaWO_4_ and Bi_2_WO_6_ show only one absorption band, while NiWO_4_ shows four absorption bands. Worth noting to report that the absorption peak of BaWO_4_ from this work was found close to what has been reported [19].

For the NiWO_4_ sample, 100 nm shift to a lower wavelength was observed as compared to the same material synthesized by the molten salt method [20]. Four bands observed from the NiWO_4_ sample at both UV and visible range (Figure 5(b)) are due to the oxidation state of the cations [37]. Cimino et al. [38] had reported that absorption bands at 1.21, 1.65-1.74, 2.00-2.11, 2.83-2.88 and 3.35 eV from Ni^2+^O_6_ are due to the transition from ^3^A_2g_ to the excited states ^3^T_2g_, ^1^E_g_, ^3^T_1g_, ^1^T_2g_, and ^3^T_1g_, respectively. Similar data were also obtained by Lenglet et al. [39] who reported the same bands at about 1.08-1.13, 1.72-1.75, 1.77-1.95, 2.71-2.79 and 2.97-3.00 eV. In the present work, four absorbance bands at 299 nm (2.97 eV), 453 nm (2.71 eV), 738 nm (1.68 eV) and 842 nm (1.47 eV) are observed; the first and second bands with high intensity are in the ultraviolet range while the third and forth with low intensity is in the blue range. The first band at 2.97 eV may be attributed to the charge transfer transition in the WO_6_ matrix. Bands at 2.71 and 1.68 eV are assigned to the forbidden electronic transition from ^3^A_2g_ to ^1^E_g_ and ^1^T_2g_, respectively. The band at 1.47 eV can be assigned to the presence of Ni^2+^O_4_ arising from Frenkel defects with dislocation of Ni^2+^ from the octahedral to tetrahedral sites. This result is in agreement with that of de Oliveira et al. [37].

Quantification of the band gap (E_g_) was carried out for all three metal tungstate samples. The band gap transition is determined from the steep shape of the spectra and the equation αhν = *A*(hν – E_g_)^m^ was employed where the absorption coefficient (α) is related to the incident photon energy (hν), *A* is constant, m is the index indicating the type of transition [38]. The nature of the electropositive ions (Ba^2+^, Ni^2+^ and Bi^3+^) seems to have small influence on the E_g_ values. It is found that E_g_ decreases according to the following sequence: BaWO_4_ > Bi_2_WO_6_ > NiWO_4_ (Table 3). The band gap of BaWO_4_ (4.60 eV) agrees well with the values reported [39,40], while the value of the prepared NiWO_4_ is significantly higher (3.05 eV) [41,42]. Ross-Medgaarden and Wachs [14] also reported the E_g_ value of wolframite NiWO_4_ as ~4.5 eV, which is higher than this finding with ligand-to-metal charge transfer (LCMT) band maximum between 247-252 and 342-344 nm, due to the distortion nature in isolated WO_6_ units.

**Table 3:** Summary of metal tungstates wavelength and band gap energy

**System**	**Wavelength (nm)**	**Eg (eV) this work**	**Eg (eV) literature**	**Ionic radius of cation A (Å)**
Bi_2_WO_6_	320.56	3.05	2.7 [43]	2.67
BaWO_4_	223.03	4.60	4.8- 5.58 [39,40]	1.42
NiWO_4_	299.03	2.97	2.28- 2.95 [41,42] 4.5 [14]	0.69

For Bi_2_WO_6_, the band gap value obtained (3.05 eV) is higher than that found by Fu et al. [43], as the E_g_ value in d° perovskites was shown to depend upon the electro-negativity of the transition metal ion, the connectivity of the polyhedral and the deviation from linearity of the M-O-M bonds. In addition, the forms of the solid samples often have strong effect on the optical properties of the material [22].

#### PL spectra

**Figure 6:**
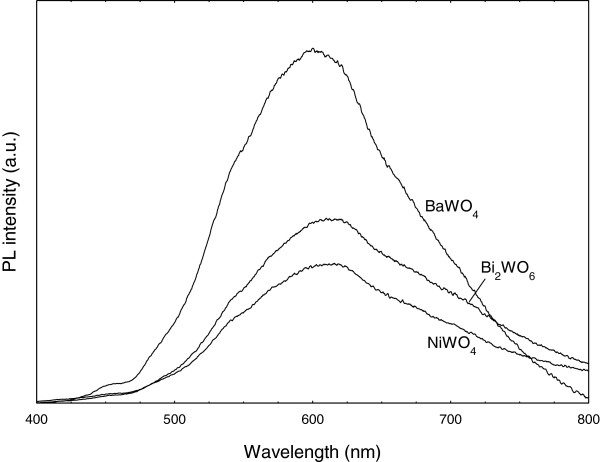
**Photoluminescence spectra of BaWO**_**4**_**, NiWO**_**4 **_**and Bi**_**2**_**WO**_**6**_** samples.**

Figure 6 shows the PL spectra of BaWO_4_, Bi_2_WO_6_ and NiWO_4_ using the excited wavelength of 325 nm. Broad blue-green emission peaks centered at ~600 nm are observed. This profile of the emission band is typical of a multiphonon process, i.e., a system in which relaxation occurs by various paths, involving the participation of numerous states. The emission peaks are attributed to the recombination of electrons in the ^1^T_2_ excited state and holes in the ^1^A_1_ ground state, specified within the [WO_4_]^2-^ excited complexes. The blue component is attributed to the regular lattice of which the emitting level comprises both metal cations (Ba, Ni and Bi) and tungstate contributions, while the green one originates from the defect centers associated with oxygen [44]. Meanwhile the broad peaks are due to the transition from the ^3^T_1_ and ^3^T_2_ excited states to the ^1^A_1_ ground state [24].

It has been reported that metal tungstates exhibit blue luminescence spectra, which is based on the radiative transition within the tetrahedral (WO_4_^2-^) group [45]. From Table 4, BaWO_4_ nanoparticles exhibit higher emission intensity than the other two samples (Bi_2_WO_6_ and NiWO_4_). The PL intensity is controlled by the number of charged transfers and surface defects. Moreover, the emission peaks obviously shift to the region of long wavelength which may be due to the particle-forming effect and the increased size of nanoparticles [46,47].

**Table 4:** **Summary of wavelength at maximum peak and peak intensity of BaWO**_**4**_**, NiWO**_**4**_** and Bi**_**2**_**WO**_**6 **_**samples**

**System**	**Intensity (a.u.)**	**Wavelength (nm)**
NiWO_4_	921	615
BaWO_4_	2600	603
Bi_2_WO_6_	1215	610

For Bi_2_WO_6_, smaller grain size also contributes considerably to high PL intensity. Similar observations were also observed by Dong Young et al. who synthesized Bi_2_WO_6_ hydrothermally and obtained higher PL intensity with the smaller crystallite size of 23 nm as calculated from XRD [48]. These phenomenon closely agrees to that reported by Quintana-Melgoza et al. [25] in which the optical response of material is largely determined by its underlying electronic properties that are closely related to its chemical or ions, atomic arrangement and physical dimension for nanometer-sized materials. Low intensity of the PL curve has been shown to be due to the oxygen atoms playing the role of electron capturers, thereby depressing the recombination process. In addition, PL intensity also depends on whether the added tungsten metal acts as an electron capturer or not. The PL curve of NiWO_4_ powder tends to shift slightly to a higher wavelength as compared to Bi_2_WO_6_ and BaWO_4_. This blue shift is observed when the dimensions of nanocrystalline particles approach the exciton Bohr radius (a_o_) due to the quantum-size effect (quantum confinement phenomenon) which can be attributed to the wider band gap [49] thus agreeing with the finding on band gap calculation in Table 3. Lee et al. [49] on discussing the effective mass model has assumed that blue shift in the band gap energy occurs due to spatial confinement of an exciton. Hence to generate a free exciton, energy higher than the effective band gap energy must be available. In the absence of additional levels introduced by defects, radiative electron-hole recombination of this free exciton should result in photon emission with energy equivalent to the band gap energy. Although there are different opinions explaining the origin of the emission bands and the nature of the optical transition is unclear, the WO_4_^2−^ complex and the slight deviation from a perfect crystal structure are believed to be responsible for the emission bands.

## Conclusions

Three different phases of metal tungstates of BaWO_4_ (scheelite), NiWO_4_ (wolframite) and Bi_2_WO_6_ (perovskite layer) were successfully prepared by the simple and economical sucrose-templated method. The highest surface area (20.06 m^2^g^-1^) contributed by of NiWO_4_ is believed to arise from higher pore distribution and less particle agglomeration due to the presence of sucrose. Raman spectra showed that the vibration modes of the products are in accordance to those of the tungstate compounds. Microstructure vibrations of three different phases of scheelite-type BaWO_4_ were shown to have less Raman active modes when compared to wolframite NiWO_4_, caused from the increased lattice symmetry while layered perovskite Bi_2_WO_6_ exhibited only four peaks involving oxygen motion, perpendicular and within the layer. Slight shifting of the detected vibration modes and that some vibration modes were not detected can be attributed to some differences in their geometries, particle sizes and nature of the products. The UV spectra revealed the highest band gap associated with BaWO_4_ followed by Bi_2_WO_6_ and NiWO_4_. Broad blue-green emission peaks in PL were detected at ~ 600 nm for all samples. The blue-shift in PL spectra is due to the quantum size effect as a result of the wider band gap. Results also showed great dependence of the PL intensity on smaller grain sizes (~ 50-80 nm) with homogenous spherical particle morphology. The materials showed promising PL results for fluorescence lamp application.

## Competing interests

The authors declare that they have no competing interests.

## Authors’ contribution

SMMZ carried out the experimental work, RY, AH, HNMEM and MND participating in the interpretation and discussion of the results. All authors read and approved the final manuscript.
